# Chicken Caecal Microbiome Modifications Induced by *Campylobacter jejuni* Colonization and by a Non-Antibiotic Feed Additive

**DOI:** 10.1371/journal.pone.0131978

**Published:** 2015-07-10

**Authors:** Alexandre Thibodeau, Philippe Fravalo, Étienne Yergeau, Julie Arsenault, Ludovic Lahaye, Ann Letellier

**Affiliations:** 1 NSERC Industrial Research Chair in Meat-Safety (CRSV), Université de Montréal, Faculty of Veterinary Medicine, Saint-Hyacinthe, Québec, Canada; 2 Swine and Avian Infectious Disease Research Centre (CRIPA), Université de Montréal, Faculty of Veterinary Medicine, Saint-Hyacinthe, Québec, Canada; 3 Groupe de recherche et d’enseignement en salubrité alimentaire (GRESA), Université de Montréal, Faculty of Veterinary Medicine, Saint-Hyacinthe, Québec, Canada; 4 National Research Council of Canada, Montréal, Québec, Canada; 5 Jefo Nutrition Inc, Saint-Hyacinthe, Québec, Canada; Charité, Campus Benjamin Franklin, GERMANY

## Abstract

*Campylobacter jejuni* is an important zoonotic foodborne pathogen causing acute gastroenteritis in humans. Chickens are often colonized at very high numbers by *C*. *jejuni*, up to 10^9^ CFU per gram of caecal content, with no detrimental effects on their health. Farm control strategies are being developed to lower the *C*. *jejuni* contamination of chicken food products in an effort to reduce human campylobacteriosis incidence. It is believed that intestinal microbiome composition may affect gut colonization by such undesirable bacteria but, although the chicken microbiome is being increasingly characterized, information is lacking on the factors affecting its modulation, especially by foodborne pathogens. This study monitored the effects of *C*. *jejuni* chicken caecal colonization on the chicken microbiome in healthy chickens. It also evaluated the capacity of a feed additive to affect caecal bacterial populations and to lower *C*. *jejuni* colonization. From day-0, chickens received or not a microencapsulated feed additive and were inoculated or not with *C*. *jejuni* at 14 days of age. Fresh caecal content was harvested at 35 days of age. The caecal microbiome was characterized by real time quantitative PCR and Ion Torrent sequencing. We observed that the feed additive lowered *C*. *jejuni* caecal count by 0.7 log (p<0.05). Alpha-diversity of the caecal microbiome was not affected by *C*. *jejuni* colonization or by the feed additive. *C*. *jejuni* colonization modified the caecal beta-diversity while the feed additive did not. We observed that *C*. *jejuni* colonization was associated with an increase of *Bifidobacterium* and affected *Clostridia* and *Mollicutes* relative abundances. The feed additive was associated with a lower *Streptococcus* relative abundance. The caecal microbiome remained relatively unchanged despite high *C*. *jejuni* colonization. The feed additive was efficient in lowering *C*. *jejuni* colonization while not disturbing the caecal microbiome.

## Introduction

*Campylobacter jejuni* is the pathogen that causes campylobacteriosis, an acute gastroenteritis in humans [1]. In industrialized countries, *C*. *jejuni* is the most frequent agent associated with bacterial foodborne infections [2, 3]. A major source of infection is the consumption of undercooked chicken meat or the mishandling of raw contaminated chicken meat products [4].

It is no surprise that chicken has been identified as a major reservoir of this pathogen [5] since *C*. *jejuni* can colonize the chicken gastrointestinal tract in large numbers, frequently at levels higher than 10^6^ CFU/g of caecal matter [6]. *C*. *jejuni* can be detected in a chicken flock at around 14 days of age and all chickens can be colonized by the end of rearing in a positive flock [7]. Experimentally, it was shown that a low dose (less than 100 CFU) of *C*. *jejuni* is sufficient for a bird’s full colonization [8]. *C*. *jejuni* mainly colonizes the chicken caecum and and is primarily presents in the mucous layer [9, 10]. This bacteria is generally recognized as having a near-commensal relationship with their chicken hosts [11], as only scarce studies have ever reported possible detrimental health effects associated with *C*. *jejuni* colonization [12]. It is still unknown how *C*. *jejuni* affects the ecology of the chicken gut.

Modern sequencing approaches [13, 14] are being used to actively characterize the chicken gut microbiome. The caecal microbiome is mainly colonized by members of the *Firmicutes*, *Bacteriodetes*, and *Proteobacteria* [15–18]. Many of these microorganisms are thought to play an important role in chicken health, in particular, as crucial agents in the degradation of complex oligo-saccharides and the production of short-chain volatile acids [18]. The chicken intestinal microbiome matures as the chicken ages [19] and can react to numerous conditions such as feed formulations or the presence of pathogens [20–23]. Despite all these advances, *C*. *jejuni* interactions within the caecal microbial community are poorly characterized.

Decreasing *C*. *jejuni* levels at the farm would be an optimal way to reduce the incidence of human campylobacteriosis acquired from chicken-associated sources [5]. Despite active research, no control method has yet been successfully and commercially implemented in the chicken industry to control this pathogen. While *C*. *jejuni* negative chicken lots can be achieved through tremendous biosecurity efforts [24], taking this route may not be feasible for the vast majority of chicken farms. One possible approach to reduce *C*. *jejuni* levels in the chicken gut is the inclusion of additives in bird feed that could lower *C*. *jejuni* caecal colonization by either direct action or by the modulation of the chicken caecal microbiome. Some essential oils and short-chain fatty acids are active *in vitro* against *C*. *jejuni* and have proven efficient, to varying degrees, at lowering chicken colonization [25]. The exact effect of these molecules on the modification of the chicken gut microbial ecology remains largely understudied.

The aim of this study was to assess the chicken caecal microbiome diversity changes induced by *C*. *jejuni* colonization. The study design was also used to evaluate the capacity of a microencapsulated feed additive, based on organic acids and essential oils, to influence caecal microbiome diversity and to reduce caecal *C*. *jejuni* levels.

## Material and Methods

### Animal model

All animal experimentations were approved by the Comité d’éthique sur l’utilisation des animaux (CEUA) of the veterinary faculty of the Université de Montréal, following guidelines from the Canadian Council on Animal Care (CCAC).

For this experiment, 4 groups were used. Chickens were inoculated or not with *C*. *jejuni* and received or not the feed additive. The two *Campylobacter* positive groups (group 1 and group 2) included 16 chickens each while the non-inoculated groups (group 3 and group 4) were composed of 8. The feed additive (Jefo Nutrition Inc., Saint-Hyacinthe, Canada), consisting of a microencapsulated mix of short chain organic acids and phenolic essential oils (the main ingredients being thymol and sorbic acid), was administered in feed at 2,000 ppm to group 1 and to group 3.

Day-old Ross 308 chickens, acquired from a local hatchery, were randomly assigned to one of the groups and immediately fed or not the feed additive. Chickens were raised in isolated concrete floored pens with wood shavings in the avian research center of the veterinary medicine faculty under a level two biosecurity confinement. Chickens had access to feed and water *ad-libitum*. Chickens were fed a standard starter diet up to 21 days of age and then received a finisher diet. Chickens were orally inoculated at 14 days of age with a mixed suspension of *C*. *jejuni* containing an equal amount of our lab strains A2008a and G2008b. These 2 strains, recovered from fresh chicken caecal content, were previously extensively characterized for their ability to colonized the chicken and compete for colonization, even in the presence of essential oils given as a feed additive [26, 27]. Each chicken received 10^4^ CFU of each strain. This inoculum was obtained by suspending in buffered peptone water (LabM, Heywook, UK) an overnight culture of both strains grown on blood agar (Fisher Scientific, Ottawa, Canada). The inoculum concentration was validated by direct enumeration on mCCDA plates (Oxoïd, Ottawa, Canada). At 35 days of age, chickens were anesthetised by electronarcosis before being ethically killed by exsanguination. Fresh caecal content was recovered at this time and sent on ice to the laboratory for *C*. *jejuni* enumeration or immediately frozen in liquid nitrogen for further molecular analysis.

### Campylobacter jejuni caecal content counts

For each chicken, 1g of caecal content was used for the enumeration of *Campylobacter* on mCCDA plates [28]. Caecal contents from the inoculated group were serially diluted and 100 μl of the diluted samples were plated and incubated for 48h in a microaerobic atmosphere (Oxoid) at 42°C prior to enumeration [29].

### Caecal content DNA extraction

A 250 μg aliquot of the frozen caecal content was added to 500 μl of lysis buffer (Tris-HCl 500 mM pH 8, EDTA 100 mM pH 8, NaCl 100mM, SDS 1% (w/v)) and to 500 mg of 0.1mm glass beads. A MP Biomedical FastPrep instrument was used to mechanically lyse the samples, using 2 runs of 30 seconds at maximum speed. Samples were kept on ice between both runs, heated to 95°C for 10 minutes, then put back on ice. Debris and glass beads were removed by centrifugation at 10,000 g for 5 minutes. The DNA was extracted from the supernatant using a standard phenol/chloroform DNA extraction [30]. The final samples were quantified and analyzed by Nanodrop (ND 1000). Samples with ratios over 1.8 for absorbance of 260 nm/280 nm or 260 nm/230 nm were kept. DNA was stored at -20°C for further analysis.

### Real time quantitative PCR of specific bacterial populations

Real time PCR was used to monitor some specific bacterial populations regularly used as indicators in classic analysis of the intestinal microbiome: total *E*.*coli*/*Shigella* [31], *Clostridium perfringens* [32], Lactobacilli [33], Enterobacteria [33], and *Bifidobacterium* [34]. Standard curve for all QPCR was constructed with known copy numbers of diluted PCR templates. For each QPCR reaction, 50 ng of DNA was amplified in a Roche LC96 Real Time PCR using Evagreen master mix (Montréal biotech, Montréal, Canada) in a 20 μl volume. Results were expressed as the log of target gene per 10 ng of amplified DNA.

### Ion Torrent 16S rRNA gene sequencing

For a broader and detailed view of the possible microbiome modulation induced by *C*. *jejuni*, 16S rRNA gene sequencing (V2-V3 region) was performed using Ion Torrent sequencing using forward primer TACGGRAGGCAGCAG and reverse primer ATTACCGCGGCTGCTGGC [14]. For 8 chickens per group, selected according to their *C*. *jejuni* status, 20 ng of DNA was pre-amplified using a MID forward primer (0.5 μM) and a universal reverse primer (0.5 μM) in a final 50 μl reaction using Platinum PCR Supermix polymerase (Invitrogen, Burlington, Canada). The amplification was carried out for 25 cycles with a denaturation step at 95°C for 30 s, an annealing step at 55°C for 30 s, and an elongation step of 45 s at 72°C. PCR products (approximately 190 pb) were purified by gel electrophoresis using the PureLink Quick Gel Extraction Kit (Invitrogen). Purified amplicon were pooled and sequenced according to Sanschagrin and Yergeau [14].

### Ion torrent data analysis

All sequencing data were analyzed [35] using Mothur v1.33 [36], based on the standard operative procedures available online, with the modifications below. When not specified, default values were used for Mothur commands.

Sequences were first trimmed using the trim.seqs command and using a qaverage score of 20, a minimum length of 100 nucleotides, and a window of 35 nucleotides. The forward primer was also kept.

For the relative abundance analysis, the sequences were then classified using a cut off of 51 percent similarity and the Greengenes database

For diversity analysis, sequences were further processed. The unique.seqs command was used to lower the computer processing load needed for analysis. Sequences were aligned against the Greengenes alignment file, using flip = true and a ksize of 6. Badly aligned sequences were removed using the screen.seqs command with the optimization set to the start of the aligned sequences and with the minimum length parameter set to 100 nucleotides. Remaining aligned sequences were filtered with the filter.seqs command. Sequences were then clustered using the pre-cluster command to correct for the effect of eventual sequencing errors diversity measurements [37]. A distance matrix was then built using the distance.seqs command with the cut-off set to 0.03. Sample coverage and diversity indexes were calculated with the summary.single command using a subsample size consisting of the lowest OTU number present in a single bird. For beta-diversity analysis, distance matrix were calculated using the distance.shared command and using the subsample = true parameter. All calculated distance matrix were visualized in a PCoA or NMDS graph. Loading and stress files were observed. The thetaYC distance matrix visualized in the NMDS plot was kept as this option was the one showing the best coverage of the total variance in the data. AMOVA and UniFrac unweighted analyses were then carried using the thetaYC phylip file. The raw sequences can be accessed via the NCBI SRA database under accession number SRA245401.

### Statistical analysis

A bird was chosen for the unit of analysis. *Campylobacter* counts between groups were compared using a Mann-Whitney test. QPCR results, relative abundance of taxon, and diversity indexes were compared across all groups with a Kruskal-Wallis test followed by Dun’s post-hoc tests. A Mann-Whitney test was then used to compare the relative abundance of taxon found in groups relative to their *Campylobacter* colonization status or to their feed being supplemented or not with the feed additive. Statistical analyses were run in GraphPad v6 (Prism, LaJolla, USA). An alpha value of 0.05 was chosen as the significance level. All presented results are statistically significant unless otherwise mentioned.

## Results

### Campylobacter counts in caecal content

**Fig 1:**
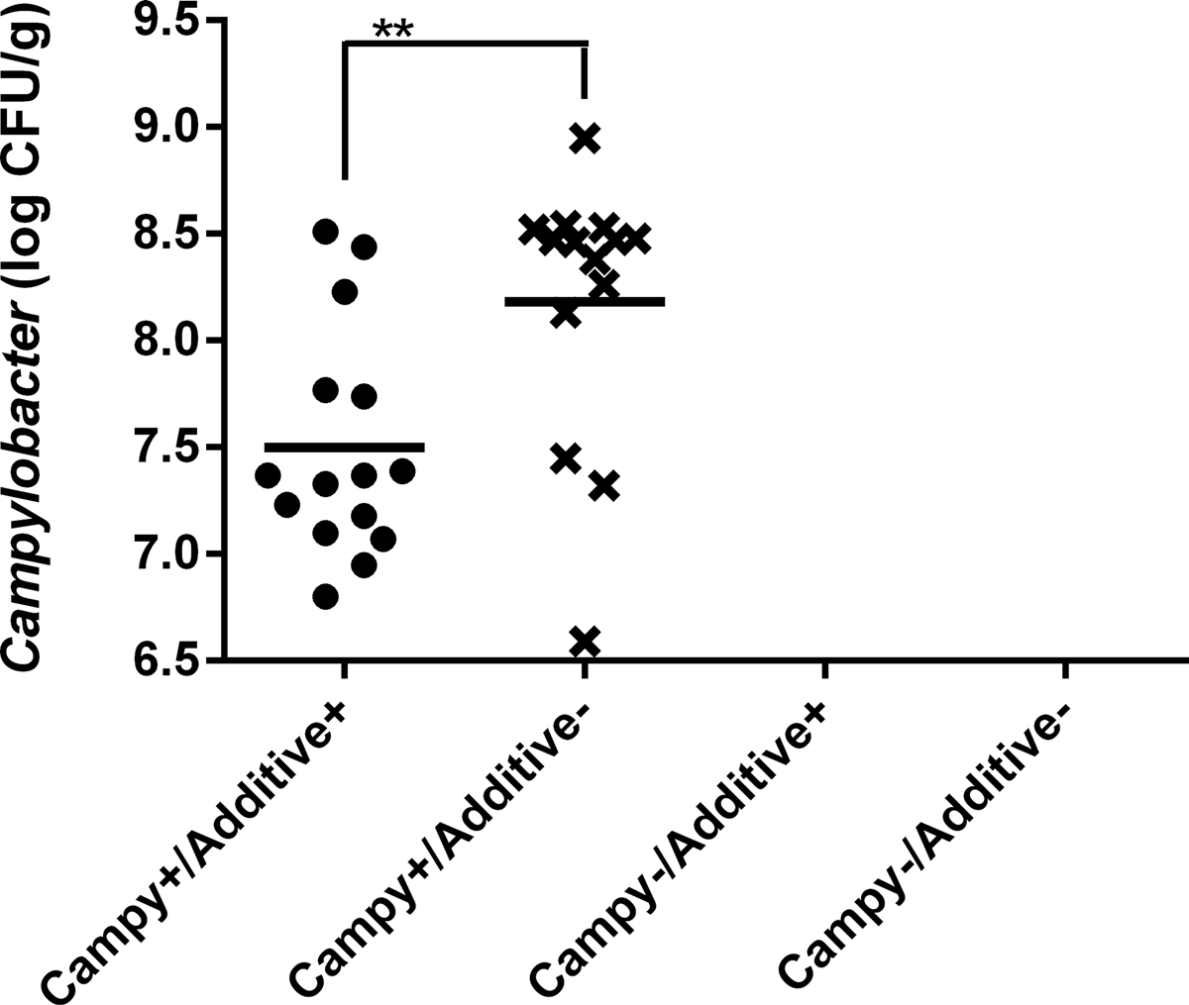
*Campylobacter* caecal counts in chickens at 35 days of age. No *Campylobacter* could be detected for the Campy- groups; each point represents the caecal content of a single chicken; horizontal bars illustrate the mean for each group; insufficient caecal matter was recovered from some chicken to allow the enumeration of *C*. *jejuni*; ** indicates p<0.01.

No *Campylobacter* was found in the non-inoculated group (Fig 1). *Campylobacter* counts for the feed additive group were lower by 0.7 log in comparison to the counts of the inoculated group not supplemented with the feed additive (Fig 1).

### Real time quantitative PCR of specific bacterial populations

**Fig 2:**
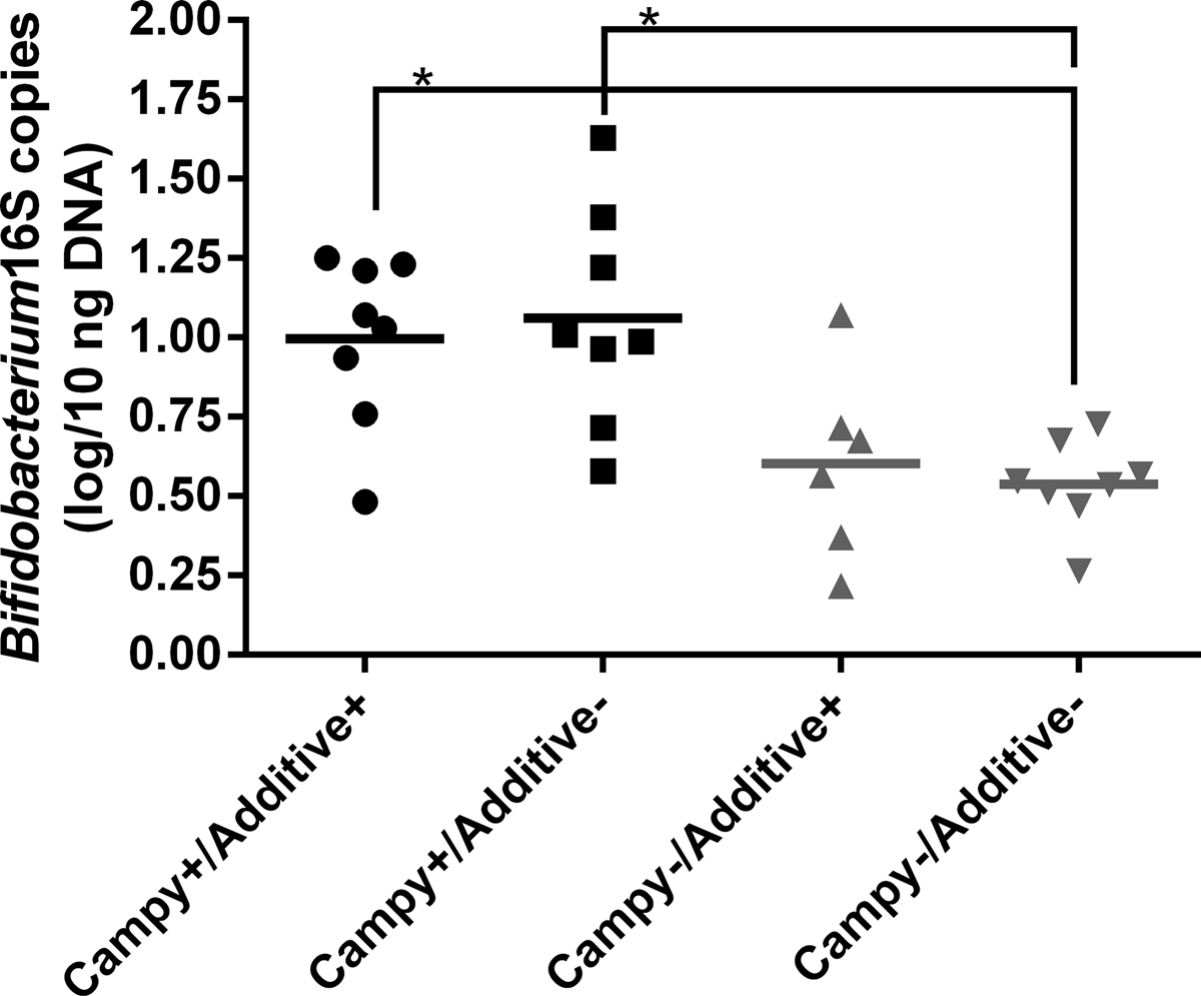
Bifidobacterium 16S copies in caecal content at 35 days of age across all chicken groups; * indicates p<0.05. Each point represents the caecal content of a single chicken; horizontal bars illustrate the mean for each group.

Standard curve efficiencies varied between 96% and 110% while all correlation coefficients (R^2^) were 1.0. A difference was seen between the groups for *Bifidobacterium* (Fig 2): detected *Bifidobacterium* 16S rRNA gene copies were higher by 0.4 log in the *Campylobacter* positive groups (p<0.001). No differences were seen for *E*.*coli*/*Shigella* (S1 Fig, *Clostridium perfringens* (S2 Fig), Lactobacilli (S3 Fig) and Enterobacteria (S4 Fig).

### 16S rRNA gene sequencing

For the Campy-/Additive + group, only 6 chicken’s samples were retained for sequencing. After trimming, a total of 178 767 sequences were retained for further analysis. Prior to the diversity analysis, the average number of OTU obtained were 3 659, 3 920, 7 525 and 5 382 respectively for group 1 to 4 while the minimum numbers of sequences respectively were 898, 1 545, 3 684 and 1 518 and the maximum numbers of OTU obtained were 6 259, 6 880, 12 260 and 8 726. Analyzed alpha-diversity parameters, using a subsample of 898 OTUs which correspond to the lowers total OTU recovered from a single chicken after sequence analysis, are presented in Table 1. Neither the colonization by *C*. *jejuni* nor the use of the feed additive modified the caecal microbiome alpha-diversity.

**Table 1:** Comparison of alpha-diversity indexes across chicken groups and according to *C*. *jejuni* colonization or to feed additive use.

Indexes	Chicken Groups				*C*. *jejuni* status		Feed additive use	
	Campy+Additive+	Campy+Additive-	Campy-Additive+	Campy-Additive-	Campy+	Campy-	Additive+	Additive-
Good’s coverage	0.85	0.86	0.86	0.85	0.85	0.85	0.85	0.86
Chao 1	701	624	620	662	663	644	667	643
Shannon	3.8	3.7	3.8	3.8	3.7	3.8	3.8	3.7
Shannon even	0.72	0.70	0.72	0.73	0.71	0.72	0.72	0.72
Simpson (1/D)	15.6	15.0	15.8	16.5	15.3	16.2	15.7	15.7

Means, based on a subsample of 898 OTU. No differences were observed between any groups.

**Fig 3:**
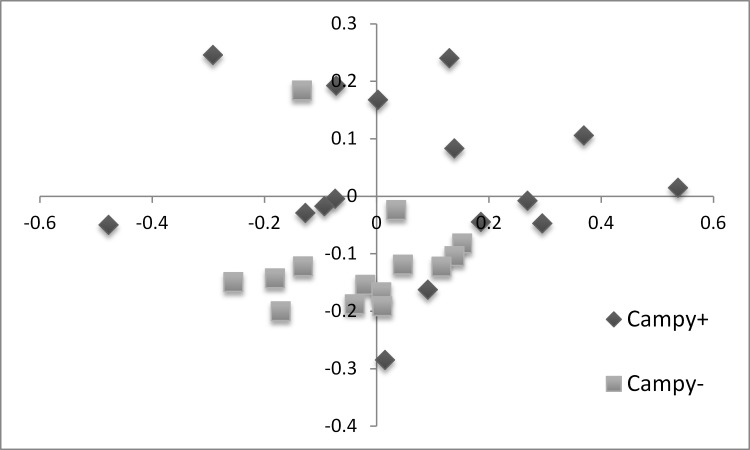
NMDS plot illustrating the chicken microbiome beta-diversity according to *C*. *jejuni* status. Each point represents a single chicken caecal microbiome; based on a subsample of 898 OTU.

**Fig 4:**
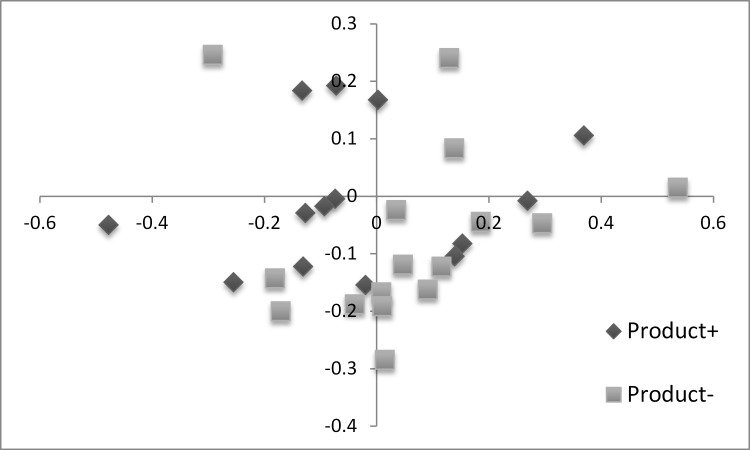
NMDS plot illustrating the chicken microbiome beta-diversity according to feed additive use. Each point represents a single chicken caecal microbiome; based on a subsample of 898 OTU.

The beta-diversity was also investigated: non-metric multidimensional scaling 2 axis plots are presented in Figs 3 and 4 while analysis of molecular variance (AMOVA) and Unifrac unweighted comparisons are shown in Table 2. The colonization of chickens by *C*. *jejuni* affected the beta-diversity while no effect from the feed additive could be observed.

**Table 2:** Beta-diversity analysis across chicken groups and according to *C*. *jejuni* colonization or feed additive use.

Groups compared		AMOVA significance (p value)	UniFrac significance (p value)
Campy+/Additive+	Campy+/Additive-	0.07	0.205
	Campy-/Additive+	0.085	0.209
	Campy-/Additive-	**0.001**	**0.045**
Campy+/Additive-	Campy-/Additive+	0.039	0.401
	Campy-/Additive-	**0.002**	0.08
Campy-/Additive+	Campy-/Additive-	0.459	0.862
Campy+	Campy-	**<0.001**	**0.001**
Additive+	Additive-	0.355	0.514

Based on a subsample of 898 OTU; *p* values returned by Mothur; p values < 0.05 are in bold.

Taxonomic analysis, based on relative abundance of the identified taxon, revealed that *C*. *jejuni* colonization induced changes in the relative abundance of detected sequences (Table 3). The main differences were observed for *Mollicutes* and *Clostridia*. No *Campylobacter* sequence was detected in the *C*. *jejuni* free chickens while sequences were obtained in all inoculated groups. Apart from a modulation of *Streptococcus* and *Dehalobacteriaceae* relative abundance, groups fed or not with the additive did not vary in terms of relative microbiome composition. Relative abundances of each taxonomic unit for each individual chicken sample are available in the supplemental material (S1 Table).

**Table 3:** Changes in the relative abundance of the caecal microbiome phylum induced by *C*. *jejuni* colonization.

Taxonomic rank	Identification	Significance (p value)	*C*. *jejuni* effect	Fold change
Genus	*Streptococcus*	0.0448	-	39
Family	unclassified *Clostridia*	<0.001	-	5
Genus	unclassified *Clostridia*	<0.001	-	5
Genus	unclassified *Lachnospiraceae*	<0.001	+	2
Genus	*Blautia*	<0.001	-	2
Family	*Mogibacteriaceae*	<0.001	+	9
Genus	unclassified *Mogibacteriaceae*	<0.001	+	9
Genus	*Anaerofilum*	<0.001	-	3
Genus	*Faecalibacterium*	<0.001	+	7
Family	unclassified *Clostridia*	<0.001	+	2
Family	*Christensenellaceae*	<0.001	-	14
Genus	unclassified *Christensenellaceae*	<0.001	-	14
Family	*Clostridiaceae*	0.0422	+	2
Genus	*Clostridium*	0.0028	+	5
Genus	*Coprobacillus*	0.0270	-	2
Phylum	*Tenericutes*	0.0011	-	2
Class	*Mollicutes*	<0.001	-	2
Order	unclassified *Mollicutes*	0.0051	-	12
Family	unclassified *Mollicutes*	0.0051	-	12
Order	*Anaeroplasmatales*	<0.001	-	12
Family	*Anaeroplasmataceae*	<0.001	-	12
Genus	*Anaeroplasma*	<0.001	-	12

In the *C*. *jejuni* effect, “-” indicates a decrease in relative abundance in birds colonized by *C*. *jejuni* while “+” relates to an increase.

## Discussion

In this study, we investigated the modification of the chicken caecal microbiome induced by the colonization of chickens by *C*. *jejuni*. We also tested the ability of a chicken feed additive to decrease the colonization level of this important foodborne pathogen and we monitored any associated modification of the chicken caecal microbiome. In this study’s experimental conditions, chickens were highly colonized by *C*. *jejuni* and the use of the feed additive lowered the caecal *C*. *jejuni* count by 0.7 log CFU/g.

In other studies, colonization levels of *C*. *jejuni* in broiler chickens were shown to be decreased by essentials oils or organic acids [38, 39] or to remain unaffected [40]. A synergistic effect on *C*. *jejuni* was also observed when both organic acids and botanicals were used as a feed additive [39]. It has been determined that a reduction of the *C*. *jejuni* chicken carcass contamination by 2 log would reduce the human campylobactersiosis risk by 30 [5]. Lowering the overall *C*.*jejuni* caecal load in chicken prior to slaughter is a good step toward this goal.

We observed a conservation of caecal microbiome alpha-diversity during either high colonization by *C*. *jejuni* or the use of the feed additive. Similar effects were also observed with *Salmonella* Enteritidis colonization in young chickens [41]. This study’s results strongly suggest that *C*. *jejuni* may become an important member of the chicken caecal microbiome without altering caecal alpha-diversity. In many other studies [19, 41], conditions tested also did not affect caecal microbiome alpha-diversity, not even necrotic enteritis [42], a severe chicken gut disorder. In another study, a severe necrotic enteritidis could be associated with changes in alpha and beta diversity [21]. Overall, based on our results and on these studies, it appears that only some drastic event that alters the number of ecological niches available to different bacterial species could modify the alpha-diversity of the chicken caecal microbiome.

In this study, beta-diversity of the chicken’s microbiome was also investigated. In our study conditions, NMDS plots highlighted differences between the birds microbiome composition, even in birds belonging to the same group. This was also reported in another study [42]. Nevertheless, the visual analysis of the NMDS graph suggested that the caecal microbiome beta-diversity was moderately affected by *C*. *jejuni* colonization and not by the additive. This was confirmed by the AMOVA and UniFrac analyses and was clearly reflected by what was observed when comparing the taxonomic rank relative abundances. The moderate but significant beta-diversity change induced by *C*. *jejuni* was quite unexpected—prior to the study we hypothesized that a high level of colonization would bring pronounced changes to the caecal microbiome ecology. In an early DGGE experiment, *C*. *jejuni* colonization was shown to affect the development and complexity of the microbial communities of the caeca over 17 days of age, in a day-old *C*. *jejuni* model [43]. More recently, another study observed in change in bacterial diversity associated with *C*. *jejuni* caecal presence in chicken without being able to clearly identify any significant changes in the microbiome composition [44].

In a recent experiment [45], *Campylobacter* carriage, assessed for birds originating from different farms and production types, was associated with moderate modulations of the caecal microbiome in birds of 56 days of age, sampled at slaughter houses. This study reported an increase in *Streptococcus* and *Blautia* relative abundance while a decrease was observed in the present study. The Kaakoush study also shown an association of *C*. *jejuni* presence with the relative abundance of taxon (*Escherichia*, *Alistipes*, *Enterococcus*, *Bacteroïdes*, *Shigella*, *Gallibacterium*, *Lactobacillus*, *Corynebacterium*, *Ruminococcaceae*, and *Enterobacter*), which was not observed in our study. Our study design is quite different than what was reported by Kaakoush. We measured chicken microbiome modifications solely induced by *C*. *jejuni* during a controlled experimental challenge with chickens fed mash feeds. Despite these disparities, some microbiome modifications were common in both studies–*Faecalibacterium* and some *Clostridium* increased their relative abundance when *C*. *jejuni* colonized the chicken caecum.

In this study, we confirmed the link existing between *C*. *jejuni* and *Clostridium*. In another study, a correlation was found between high *C*. *perfringens* levels (> 6 log) and *C*. *jejuni* colonization levels [46]. In our study conditions, this relative abundance increase of *Clostridium* was not due to *C*. *perfringens* levels, as demonstrated by the analysis of the *C*. *perfringens* QPCR results (S2 Fig). To explain this relationship, it was suggested that *Clostridium* organic acid production could be used by *C*. *jejuni* as an energy source [45]. These organic acids are also used as an energy source by chickens [47]. *C*. *jejuni* could also act as a hydrogen sink that would allow better growth of some *Clostridium* through increased fermentation, leading to increased organic acid production *[* 45]. This *Campylobacter-Clostridium* relationship still needs to be better documented and the exact species that could be interacting needs to be examined. In our study, a *Christensenellaceae* (closely related to *Clostridium*) relative abundance increase was also induced by *C*.*jejuni* colonization. This is a relatively new family of bacteria [48] and its involvement in chicken health is unknown.

The association of *C*. *jejuni* and *Faecalibacterium* needs to be put into perspective. *Faecalibacterium* is closely related to members of *Clostridium* cluster IV [49]. In humans, it was shown that a decrease of *Faecalibacterium prausnitzii* was associated with intestinal disorders such as colitis [50]. *Faecalibacterium prausnitzii* is also a butyrate producer [51] and is located near the epithelial cells as it attaches to the mucous layer [49]. If this can be transposed to chickens, *Faecalibacterium* could be considered to share an ecological niche with *Campylobacter* in chickens. Its ability to produce butyrate appears to be in contradiction with its positive association with *C*. *jejuni* since butyrate was reported to be detrimental to *C*. *jejuni* [52]. It is also proposed, in humans, that *Faecalibacterium prausnitzii* could modulate the production of mucins by the goblet cells [53]. If this effect is also true in chickens, we hypothesize that it could be beneficial to *C*. *jejuni* as the mucous layer is believed to interfere with the organic acid anti-*Campylobacter* effect [7]. How *Campylobacter* could positively interact with *Faecalibacterium* and the relative importance of *Faecalibacterium* for chicken intestinal health remains to be determined experimentally.

In this study, a diminution of the relative abundance of *Mollicutes* and *Anaeroplasmateles* (*Mollicutes* class) was induced by *C*. *jejuni* colonization. In past studies, it was reported that *Mollicutes* were enriched in birds affected by the intestinal disease necrotic enteritidis and thus could possibly be associated with intestinal disorders for chickens [42]. The exact role of *Mollicutes* in the chicken microbiome is still unknown.

We observed an increase in the molecular detection of *Bifidobacterium* induced by *C*. *jejuni* colonization. *Bifidobacterium* increased levels were often associated with better gut health [54] but were also reported to hinder *C*. *jejuni* [55, 56]. It is therefore impossible to conclude if the changes observed in regards to *Bifidobacterium* levels were beneficial for chicken caecal health, in this study’s conditions. This observation could only be made by QPCR and not by the Ion Torrent analysis. The QPCR detected low levels of *Bifidobacterium*, probably too low to be picked by the Ion Torrent analysis. This could indicate that subtle changes, undetected by 16S sequencing, could still be occurring during *C*. *jejuni* colonization.

For the feed additive, an interesting decrease of *Streptococcus* relative abundance was also observed, which might be a good addition to its reduction of *C*. *jejuni* colonization since *Streptococcus* can cause diseases in chickens [57].

This study’s results suggested that the tested feed additive did not greatly imbalance the caecal microbiome. Modifications of the broilers caecal microbiome could be associated with the use of organic acids or essential oils in a previous study [58]. However, it has also been reported that essential oils were not always proven to be able to disturb the chicken caecal microbiome even when some beneficial effects on the bird’s health could be recorded [59]. It would also be interesting to verify the effect of the feed additive on the ileal microbiome as modifications of other segments of the chicken gut have not always been found to be reflected in the caecum [60].

## Conclusion

Based on these presented results, we conclude that *C*. *jejuni* colonization induced a moderate alteration of the chicken caecal microbiome diversity. This modification did not appear to be toward undesirable bacterial populations. This is in accordance with the fact that *C*. *jejuni* rarely causes harm to the birds when colonizing the chicken caecum. Based on these study observations, it can be concluded that the chicken caecal microbiome is stable and not extensively disturbed when colonized by foodborne pathogens such as *C*. *jejuni*. We also conclude that the feed additive used was able to significantly reduce *C*. *jejuni* colonization and that it could potentially reduce *Streptococcus* abundance in chicken caecal contents.

## Supporting Information

S1 Fig *E*.*coli*/*Shigella ycc*T copies in caecal content at 35 days of age across all chicken groups. (TIF)

S2 Fig *Clostridium perfringens* 16S copies in caecal content at 35 days of age across all chicken groups. (TIF)

S3 Fig Lactobacilli 16S copies in caecal content at 35 days of age across all chicken groups. (TIF)

S4 Fig Enterobacteria 16S copies in caecal content at 35 days of age across all chicken groups. (TIF)

S1 Table Relative abundance of each taxonomic unit found within each individual chicken sample. (XLSX)
